# Development of a System Measurement Model of the Brazilian Hospital Accreditation System

**DOI:** 10.3390/ijerph15112520

**Published:** 2018-11-11

**Authors:** João Éderson Corrêa, João Batista Turrioni, Carlos Henrique Pereira Mello, Ana Carolina Oliveira Santos, Carlos Eduardo Sanches da Silva, Fabrício Alves de Almeida

**Affiliations:** 1 Institute of Industrial Engineering and Management, Federal University of Itajubá, Av. BPS, 1303, Itajubá, Minas Gerais 37500-903, Brazil; edercorrea@unifei.edu.br (J.E.C.); joabatu@gmail.com (J.B.T.); carlos.mello@unifei.edu.br (C.H.P.M.); sanches@unifei.edu.br (C.E.S.d.S.); fabricio.alvesdealmeida@gmail.com (F.A.d.A.); 2 Institute of Integrated Engineering, Itabira Campus, Federal University of Itajubá, Rua Irmã Ivone Drumond, 200, Itabira, Minas Gerais 35903-087, Brazil

**Keywords:** health services, accreditation, structural equation modeling

## Abstract

The purpose of this study is to develop and validate a measurement model that evaluates the Brazilian hospital accreditation methodology (ONA), based on a multivariate model using structural equation modeling (SEM). The information used to develop the model was obtained from a questionnaire sent to all organizations accredited by the ONA methodology. A model was built based on the data obtained and tested through a structural equation modeling (SEM) technique using the LISREL^®^ software (Scientific Software International, Inc., Skokie, IL, USA). Four different tests were performed: Initial, calibrated, simulated, and cross-validation models. By analyzing and validating the proposed measurement model, it can be verified that the selected factors satisfy the required criteria for the development of a structural model. The results show that leadership action is one of the most important factors in the process of health services accredited by ONA. Although, leadership, staff management, quality management, organizational culture, process orientation, and safety are strongly linked to the development of health organizations, and directly influence the accreditation process.

## 1. Introduction

### 1.1. Motivations

Accreditation is an approach established at international level and is a quality assurance system that adheres to a specific standard, approved by an accreditation body [1]. In the area of health, hospital accreditation is used as a management methodology that fosters strategic understanding involving all employees of the institution through a permanent educational process that shares principles, goals and objectives to be achieved. This multidisciplinary “productive” consensus aims to rationalize the use of resources and optimize results [2].

There has been an increase in the number of accreditation programs in the health area. Nevertheless, the way in which these methodologies are proposed by different accreditation models is one of the points that has been debated at the international level and also by the International Society for Quality in Health Care (ISQua) [3,4,5,6]. In this context, hospital accreditation is becoming recognized as an important approach in quality assurance processes, although few studies seek to analyze its performance and influence in organizations that adopt this type of methodology [7,8,9,10,11,12,13,14].

In Brazil, this process began to be developed in the late 1970s, when the Ministry of Health established a series of norms and ordinances that addressed the improvement of the quality of health services. In the 1980s, the Pan American Health Organization (PAHO) established standards for hospital services in Latin America that inspired the development of the hospital evaluation instrument in Brazil, created in the late 1990s, with the aim of promoting improvement of the quality of care provided by hospitals to the patient/client

In 1995, the Ministry of Health presented a project for accreditation in Brazil that resulted in the first “Brazilian Manual of Hospital Accreditation” in 1998. The following year, the National Accreditation Organization (ONA) was created, a nonprofit civil society recognized by the Ministry of Health and the National Health Surveillance Agency (ANVISA). ONA has the functions of coordinating the Brazilian System of Hospital Accreditation, defining systematic evaluation, developing quality standards, and training multipliers [15].

The standard established by the Brazilian Manual is based on three levels of complexity: Level 1—accredited, which refers to the existence of processes aimed at ensuring patient safety; Level 2—full accreditation, which refers to the integrality of the management, involves the monitoring of security barriers, processes and protocols implemented, critical analysis of care process controls, action plan establishments, improvement plans, and intersectorial interaction; and Level 3—accredited with excellence, which deals with the stage in which the organization has already incorporated a critical follow-up of the designed processes of its assistance results, developing improvement cycles in a systematic way, and has its decision-making process aligned with the institutional strategic planning [15].

However, despite the increasing efforts made by ONA, the health services situation in Brazil is still a concern, especially when comparing the quality of the services offered by the institutions with the quality aspects defined by ONA. There is also a great need to make public health more equitable, homogenous in the territory and capable of facing the growing challenges related to the Brazilian demographic dynamics [4,14].

On the other hand, sustainability is also about the ability of organizations to be able to withstand challenges and variations over time through continuous improvement processes [16,17]. Some studies indicate that organizations that are directly involved in the factors related to sustainability have positive results in the life cycle of the evaluated programs, in the capacity for innovation and in their financial results [17,18,19,20].

Regarding the studies found in the literature, the contribution of this work consists in the extension of the study formulated by Corrêa et al. [14]. Analyzing this study, it is verified that its objective is to identify the influence of quality management in the Brazilian accreditation ONA and in the sustainability of accredited companies. However, it is necessary to develop and validate a measurement model for the ONA accreditation methodology, which is characterized as one of the contributions of this work. By making a thorough analysis in the literature, it is possible to verify that only two papers measure hospital accreditation through the modeling of structural equations, examining factors from similar variables, as well as considering relevant items in the accreditation process [21,22]. Nevertheless, none of these studies were done for the ONA accreditation method, which is unique in its application and context. The theoretical models found in the literature allowed the researchers to elaborate a new theoretical model that verify the hypothesis of the relationships between the variables that the data could confirm (or not). Although studies have been done to investigate the influence of ONA accreditation on quality control [1], no study has proposed and validated a measurement model, which considers the relationship of several factors in the ONA accreditation methodology. Therefore, this research is also important for the contribution of scientific knowledge in the literature on this subject.

Thus, in this scenario, this work has two main objectives: first, to identify in the literature which are the main factors that can influence the Brazilian hospital accreditation ONA, helping researchers and managers in the decision-making process related to accreditation; and second, to develop and validate a measurement model that evaluates the Brazilian hospital accreditation methodology ONA. For this purpose, a multivariate model is proposed and analyzed through the modeling technique of structural equations and four different tests were performed: initial, calibrated, simulated, and cross-validation models.

### 1.2. Literature Review

Although the assessment of accreditation performance is difficult due to its particularities, there is a consensus in the literature about the positive results of using quality practices and accreditation in hospital organizations [10,14]. The concept of accreditation was introduced in the Brazilian health lexicon more than two decades ago, however, it was only in recent years that the subject began to arouse the interests of researchers and professionals in order to measure the effectiveness of the Brazilian accreditation model. In this context, there is a need to identify the main factors (constructs) that directly affect this process, to establish relationships between them and to analyze their interactions, since these factors can play a crucial role in the Brazilian accreditation process ONA.

The constructs used in the development of this paper were selected based on a systematic review (SRL) of the current literature. This SRL was performed in different databases in order to promote a search for articles related to accreditation instruments constructed based on structural equation models. The search strategy followed the propositions established by Greenfield and Braithwaite [7]. Articles on approaches to hospital accreditation, health services, and quality measurement models were critically selected, evaluated, and analyzed. The search for articles was carried out at Emerald, Elsevier, Taylor, and Francis databases and ISI Web of Science. A total of 57 studies were selected. After the refinement of these articles, 32 articles that fit the scope of this research were selected.

According to Hair et al. [23], a good literature review is the necessary condition for the construction of a reliable model and for obtaining useful results in Structural Equations Modeling (SEM), since it involves the prioritization of the constructs found in the literature in order to substantiate and justify the purpose of the current research. The process of selecting the constructs followed the following criteria, initially, the most commonly used constructs were identified in the literature; then, the constructs that have the same objectives were grouped together, and finally, the constructs corresponding to the objectives of this research were selected. These theoretical models allowed the researchers to elaborate a new theoretical model that hypothesizes the relations between the variables that the data would confirm or not. These constructs were named as follows.

- Leadership Construct (L): Refers to the commitment of the senior management, traditionally considered one of the most powerful bodies in quality control. This force is capable of building, defending, and supporting a context that leads to high organizational performance, individual development, and organizational learning, crucial factors towards quality development inside an organization [1,9,10,13,22,24,25,26,27,28,29,30,31,32,33].
- Sustainability Construct (SU): This construct defines the organization’s ability to deal with challenges and variations that occur over longer periods of time, using a process of continuous improvement. This construct is directly linked to the adoption of quality practices that present more desirable results in the work processes, patient safety, and financial stability of the organization. It considers aspects related to the capacity an organization has to develop, maintain, and constructively promote a more positive performance in work practices, presenting constant improvements in its processes and management system [6,17,18,19,27,33,34,35].
- People Management Construct (PM): The main aspect considered in this construct is on how well the organization engages, manages and develops its work force. This construct also relates to how the organization administers its mission, strategy, and action plans in order to use all of its human potential. The capacity of an organization to evaluate the needs of its team, creating a high performance work environment is also considered in this scope. Several authors have been searching to analyze the relationship between this construct and other variables such as performance of employees [27,29,30,33,35,36,37,38], continuous improvement [10,25,29,35,36], setting goals [6], and control [6,32].
- Organizational Culture Construct (C): Refers to the “state” or set of characteristics that describe the desire to pursue a course of action focused on the final goal [9]. Organizational culture has a significant effect on the successful implementation of accreditation. Such culture is inherent of the institution and therefore is not a characteristic of its individuals [22]. Other authors have also been addressing the relationship between this construct and other variables such as organizational commitment [10,22,39], continuous education [29,30,31,40,41], performance [22,39], and environment [31,40].
- Quality Management Construct (QM): The literature indicates that health institutions employ quality practices related to customer satisfaction, process control, competitive benchmarking, and to the existence of a quality team [26,32], which relates to the existence of an integrated team focused on patient safety through well-defined processes. There have also been studies that assert the relationship between this construct and other variables such as: quality department [27,30,35], continuous improvement [10,29,32,35,36], quality of information available [27,36,41], and process quality standards [6,40].
- Process Orientation Construct (PO): The literature treats process orientation as activities related to the existence of well-defined processes in all activities of an institution with focus on health sectors, personnel management, and in senior management. Protocols, internationally standardized processes, cooperation, measurement, data analysis, and continuous improvement are addressed in this construct. Process orientation involves points related to constructs that were identified in the literature. Some authors have been analyzing the relationship between this construct and other variables such as processes [1,10,34,40] and process management [1,13,29,30,32,42].
- Safety Construct (S): This construct deals with a fundamental factor, especially when dealing with Brazilian hospital services. In the literature, safety is referred to as the common perceptions the members of an organization have regarding their policies and safety practices, which are directly influenced by senior management. Recently, health care operation management researchers such as McFadden, Stock, and Gowen [25] have developed empirical tests on a theoretical model that evaluates the impact of safety in service quality. Several authors have approached this construct in different aspects such as climate safety practices, patient safety, safety management, senior management engagement, and interactivity [22,25,40].
- Accreditation Construct (A): This construct refers to the methodology used in Brazilian accreditation, a voluntary evaluation method that aims to ensure the quality of health services through patterns previously defined by the Brazilian Method of Hospital Accreditation (ONA). This construct refers to how an organization uses accreditation to improve the quality of their services, or how an organization establishes its processes based on a set of predetermined patterns [30].

## 2. Materials and Methods

### 2.1. Research Hypotheses

The question of global investigation of this paper refers to the measurement of factors that directly affect the hospital accreditation process ONA. It is specifically a research that adopts an interorganizational approach through the use of structural equation modeling (SEM) techniques.

**Figure 1:**
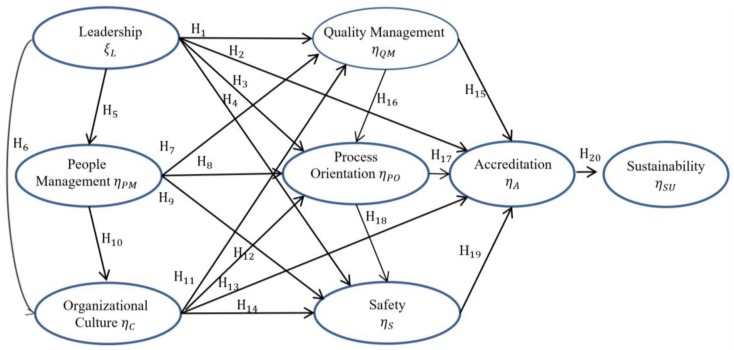
Basic conception of the model.

According to Marôco [43], the SEM technique uses hypotheses that are identified through literature reviews. Therefore, the selection of factors or constructs used in the development of this model was performed based on the SRL (presented in the previous topic) and the process of conceptualization revealed that the main constructs found were included in the proposed model. The development of the research hypothesis represents the interpretation of a series of hypothetical relations of cause and effect between the variables and its objective is to verify the existence of correlations between variables of the proposed model. The directions of the arrows indicate the causal directions between one variable and the other [44]. Figure 1 presents the hypotheses established in the model.

After building the model by means of a path diagram, the model is transformed into a system of equations. According to Bollen [45], the equations referring to a structural model with latent variable, developed by the SEM technique, is described by Equation (1):

(1) η=Bη+Γξ+ζ

where

η = η_1_,…, η_1m_

are the random vectors representing the dependent variables (endogenous)

$$B=\left[\begin{array}{cccc} 0 & \beta_{12} & \vdots & \beta_{1r}\\ \beta_{21} & 0 & \vdots & \beta_{2r}\\ \vdots & \vdots & \vdots & \vdots \\ \beta_{r1} & \beta_{r2} & \vdots & 0 \end{array}\right]$$

is the matrix (r×r) of the coefficients of η in the structural model with $\beta_{ii}=0$

$$\Gamma =\left[\begin{array}{cccc} \gamma_{11} & \gamma_{12} & \vdots & \gamma_{1s}\\ \gamma_{21} & 0 & \vdots & \gamma_{2s}\\ \vdots & \vdots & \vdots & \vdots \\ \gamma_{r1} & \gamma_{r2} & \vdots & \gamma_{rs} \end{array}\right]$$

is the matrix (r×s) of the coefficients of x in the structural model; and

$$\zeta =\left[\begin{array}{c} \begin{array}{c} \zeta_{1}\\ \zeta_{2} \end{array}\\ \vdots \\ \zeta_{r} \end{array}\right]$$

is the vector (r×1) of the r residuals or errors of the structural model.

According to Hair et al. [46], the use of SEM has an advantage over other estimation techniques since it considers the measurement error. This measurement error is expressed as a function of the regression coefficient according to Equation (2).

(2) $$\beta_{yx}=\beta_{s}+\rho_{x}$$

where:

$\beta_{yx}$ = Observed regression coefficient.
$\beta_{s}$ = True regression coefficient.
$\rho_{x}$ = Reliability of the independent variable.

The equations of the measurement model for the constructs selected for this research were developed based on the equations previously established. Equations (3)–(9) were developed using the structural modeling techniques proposed by Marôco [43], Anderson and Gerbing [47], and Kline [44], and admitting the premises established by Bollen [45]. The following equations refer to the structural model that evaluates the influence of constructs on the accreditation of institutions accredited by the methodology developed by ONA.

(3) $$\eta_{\mathrm{PM}}=\gamma_{PML}\times \xi_{L}+\zeta_{PM}$$

(4) $$\eta_{C}=\gamma_{\mathrm{CL}}\times \xi_{L}+\beta_{CPM}\times \eta_{\mathrm{PM}}+\zeta_{C}$$

(5) $$\eta_{\mathrm{QM}}=\gamma_{\mathrm{QML}}\times \xi_{L}+\beta_{QMPM}\times \eta_{PM}+\beta_{QMC}\times \eta_{C}+\zeta_{QM}$$

(6) $$\eta_{\mathrm{PO}}=\beta_{POQM}\times \eta_{\mathrm{QM}}+\gamma_{POL}\times \xi_{L}+\beta_{POPM}\times \eta_{\mathrm{PM}}+\beta_{POC}\times \eta_{C}+\zeta_{PO}$$

(7) $$\eta_{S}=\beta_{SPO}\times \eta_{PO}+\gamma_{SL}\times \xi_{L}+\beta_{SPM}\times \eta_{\mathrm{PM}}+\beta_{SC}\times \eta_{C}+\zeta_{S}$$

(8) $$\eta_{A}=\beta_{AQM}\times \eta_{QM}+\gamma_{AL}\times \xi_{L}+\beta_{APO}\times \eta_{\mathrm{PO}}+\beta_{AC}\times \eta_{C}+\beta_{AS}\times \eta_{s}+\zeta_{A}$$

(9) $$\eta_{\mathrm{SU}}=\beta_{SA}\times \eta_{A}+\zeta_{SU}$$

Six hypotheses were considered that test the influence of leadership over the following constructs: Quality management (H1), accreditation (H2), process orientation (H3), safety (H4), personnel management (H5), and organizational culture (H6). These hypotheses have already been confirmed through studies developed by Kunkel, Rosenqvist, and Westerling [1], Claver, Tarí, and Molina [13], Kou, Lee, and Wei [48], Li [24], McFadden, Stock, and Gowen [25], Meyer and Collier [26], and Lee, Suh and Han [28]. Hypotheses H7–H10 were based on the work developed by Awuor and Kinuthia [10], Choi et al. [36], Faye et al. [31], and Li [24] and test the relationship between personnel management over the constructs: quality management, process orientation, safety, and organizational culture. Hypotheses H11–H14 consider the relationship between the organizational culture construct and the following factors: quality management, process orientation, accreditation, and safety [10,22,31,40]. The relationships between quality management and the following constructs: accreditation and process orientation are described by hypotheses H15 and H16 and have been approached by Cheng [41], Choi et al. [36], El-Jardali et al. [30], Gowen et al. [32], and Meyer and Collier [26]. The influence of process orientation on accreditation and safety are presented through Hypotheses H17 and H18 [1,13,30,32,42]. Hypothesis H19 verifies the relationship between safety and accreditation [25,40].

To develop and validate a measurement model that evaluates the Brazilian hospital accreditation methodology (ONA), its necessary to test the hypotheses established in the SEM method. Therefore, the data were collected through a survey exploratory research in all health organizations accredited by ONA in Brazil. For the elaboration of the questionnaire, 59 questions found in the literature tools were selected and this activity was developed together with the ONA superintendence. All the questions used in the questionnaire were elaborated always having the same sense, using Likert scale of 1 to 7, with variations from totally disagree to totally agree.

After the development of the initial questionnaire, the next step was the evaluation of the tool made by specialists. The feasibility of the questionnaire was made through the evaluation of two groups of experts, following the propositions established by Forza [49]. The first group of experts was composed of four professors, of which three were professors of Brazilian universities with extensive experience in quality management and survey, as well as one foreign university professor with extensive experience in accreditation and clinical engineering. The second group was composed of five experts in the area of accreditation who act directly as evaluators of the ONA methodology. The objective of this step was to evaluate whether the instructions, as well as the questions of the questionnaire, were established in a clear and easily understood manner. After the review of the experts, the final questionnaire with 51 questions was developed and considered appropriate to meet the objectives of this research. The relationship of the variables of each construct and its measurement items (questionnaire questions) are presented in Table 1. The complete questionnaire is presented in Supplementary Materials.

The survey was developed and validated based on the study developed by Forza [49]. The stages regarding the development of the model were based on propositions established by Anderson and Gerbing [47] and Hair et al. [50].

### 2.2. Data Collection and Management

The data used in this research were collected by ONA, through an agreement signed between ONA and the Federal University of Itajubá (Unifei), in which ONA was responsible for delivering the research and sending the electronic questionnaire to the e-mail address of all the institutions accredited by ONA, which occurred in two shipments between August and September 2016. The collection, treatment, and analysis of the data were the responsibility of the researchers directly linked to this research. The questionnaire was sent to all 515 accredited institutions and the response rate was 49.41%. It was verified through initial analysis that there was no data codification error.

The outlier analysis was conducted based on a discriminant analysis, using the Mahalanobis distance to classify the observations in their groups predicted using the Minitab^®^ software (Minitab Inc., State College, PA, USA). It was found that some outliers presented themselves in different questions and that the respondents had no relation between them. For this reason, it was decided to exclude these outliers from their respective questionnaire, but the remaining responses to this questionnaire were maintained. This arrangement was adopted with the aim of eliminating the flaws that may be caused in the analysis of the sample or population. In the individual analysis of these outliers, it was also found that all of them presented errors of the type “execution error”, that is, for unknown reasons, 18 respondents classified the statements with the same value most respondents assigned the value 7 or 6 to all the questions. In this sense, the questionnaires that presented this kind of response were excluded. Such an arrangement was adopted since the presence of atypical data had large residuals and can exert influence on the adjusted model and because they present characteristics different from the other observations and do not represent the population. The outlier evaluation analysis pointed towards the existence of 24 outliers, and this set was treated consistently so that it did not affect the dataset that was used in this research.

The missing values analysis was performed using the Listwise software (complete case), eliminating eighteen surveys, which correspond to 2.77% of the dataset.

The reliability of the sample was calculated by measuring the internal consistence of the data (Cronbach’s alpha = 0.9603) which is considered satisfactory for the analysis of the questionnaire.

### 2.3. Evaluation of the Measurement Model

Once the information required to generate the study was gathered, the evaluation procedures of the measurement model were developed according to Alwin [51], Hair et al. [50], Kline [44], and Marôco [43]. This measurement is evaluated in two stages: the first stage consists of an evaluation for the exogenous construct and the endogenous constructs are evaluated in stage two. The adjustment quality of the model is measured using an exploratory factorial analysis and by measuring the outcome of the following tests, dimensionality, convergent validity, reliability, and discriminant validity [52,53].

A very small outcome was found when performing the Barlett’s sphericity test (Sig—*p* = 0.000). On the other hand, the Kaiser–Meyer–Olkin test (KMO > 0.857) presented significant values for all eight constructs, confirming the extraction technique as adequate for this dataset. The descriptive statistics were developed using the factor extraction technique through the principal components method, without rotation, developed based on the analysis of the covariance matrix [47].

After performing factorial analysis, it could be verified that six of the eight constructs (L, SU, PM, C, QM, and S) could be represented by 2 or 3 factors with an explanatory power of >75% of the total variance. In this way, it was found that its variables (presented in Table 1) could be grouped into factors (“Factors” in Table 1) and thus reduce the dimensionality of these constructs. This did not happen in the accreditation and process orientation constructs which could be represented by only one factor.

The commonality analysis obtained for each of the measurement items presents values greater than 0.5. It was possible to verify which questions are related forming their respective factors, and these were compared to the studies identified in the literature.

The verification of the multicollinearity of the data was performed by calculating the Variance Inflation Factor (VIF), and it could be observed that the values indicated for each variable were smaller than 5, indicating that the multicollinearity effect is not evident.

However, the evidence of convergent validity is reinforced when the results of the estimates (*γ*) > 0.50 and the results of the obtained values (*t*-value) are verified, suggesting a good general adjustment of the model considering the propositions established by Steenkamp and Van Trijp [54].

The dimensionality test for the constructs used in this model were performed based on the results obtained by the confirmatory factorial analysis developed using the LISREL^®^ software (Scientific Software International, Inc., Skokie, IL, USA). The statistical adjustments presented values within the generally accepted boundaries [55]. This result is confirmed by the absolute values above *R*^2^ > 2.58.

The convergent validity tests are derived from the factorial loads of the measurement items of each construct, which should present significant values greater than 0.50 [47,50]. Considering the results obtained for the *γ* coefficients, for the standardized solution and *t*-values, it can be concluded that the measurement models cover enough evidences for the convergent validity. The reliability tests consisted in determining the Cronbach’s Alpha which presented results >0.8, which confirmed the reliability of the constructs. These results can be verified in Table 1.

Based on the correlation matrix analysis and the extracted average variance, the evidence of discriminant validity is supported according to the premises established by Steenkamp and Van Trijp [54].

### 2.4. Evaluation of the Structural Model

After careful analysis of the measurement model describing the latent values, the results were grouped in a specification model. The model was estimated by the maximum verisimilitude method, using version 9.2 of the LISREL software. It was verified, using the initial model, that the hypotheses H2 (L and A = 2.72), H4 (L and S = 0.31), H5 (L and PM = 0.91), H7 (PM and QM = 1.00), H8 (PM and PO = 1.00), H10 (PM and C = 1.08), H12 (C and PO = 0.23), H14 (C and S = 1.00), H16 (QM and PO = 0.98), H17 (PO and A = 1.41), H18 (PO and S = 0.10), H19 (S and A = 3.37), and H20 (A and SU = 0.82) provide positive and strong estimates, showing that they played an important role in the construction of the model. On the other hand, hypotheses H1 (L and QM = −0.18), H3 (L and PO = −1.26), H6 (L and C = −0.007), H9 (PM and S = −0.93), H11 (C and QM = −0.49), H13 (C and A = −0.53), and H15 (QM and A = −2.08) provide negative estimates, evidencing that these relations are independent. This result could have been influenced by several factors. Firstly, the difference between the institutions such as: size of the institution, level of accreditation, location, type of organization as well as the internal organizational culture should be considered.

In addition, institutions with an excellent level of accreditation (123 responses) have a higher average response when compared to the other levels. The sample size of each level of accreditation: 123 organizations accredited with excellence; followed by 89 organizations fully accredited; and 79 accredited organizations. In this sense, it is possible to affirm that incoherencies between responses in different levels can affect the quality of the dataset. Elements related to relative questions should also be considered since they can suffer interferences such as poor comprehension of the survey and intensity of contact between the participants and the research administrators. Also, the interest on the outcome of the research can cause biased results [56].

According to Marôco [43], obtaining results values different from zero through the measurement variables can occur due to several factors such as: lack of similarity between responses for the same questions, due to the existence of outliers or by the lack of definition of these variables, which can interfere in the results. In this study, several fixation tests were developed in the calibrated method, in order to obtain the desired results.

Still according to Marôco [43], there is no single rule to evaluate an adjustment model. However, some tests should be done until it shows convergent results with the predicted indicators. To accomplish this step, it was chosen the combination of the factors of the measurement model that presented better quality of the adjustment of the modeling of the observed data. Initially, the negative hypotheses were withdrawn one by one and the model tested in each of these exclusions. Then, some variables removed were added again.

Other actions were also developed: the alternation of the parameters of the variables, the variables that had fixed parameters were parameterized again in free format or vice versa. Based on several tests, we found the respecified model that contains appropriate adjustments. The results showed positive standardized significance, and *t*-values that comply with the limits indicated in the literature. The results of this model can be verified in Table 2.

Before evaluating the initial and calibrated model, the development of a new model was proposed based on the simulated data, with the objective of verifying if the proposed model performs well regarding a wide range of responses.

The simulated model is presented to validate the proposed model and to verify the behavior of the model fit in a disturbance of the data. That is, to verify if the model proposed in this research is consistent and validate it—which is one of the objectives of this paper.

The dataset was obtained from a sample covariance matrix built from the average and covariance vectors, which were based on the original dataset of the survey. The values of the main diagonal are equal to the variance vectors. Generally, the variance between two questions is the quotient between the correlation and the square root of the product between the two variances, while the remaining values of the matrix are calculated according to Equation (10):

(10) $$\mathrm{Covariance}\;\mathrm{matrix}\;\mathrm{values}=r\times \sqrt{\left(s_{1}^{2}\times s_{2}^{2}\right)}$$

The remaining values that make up the covariance matrix are obtained analogously, by getting the simulated correlations for each set of questions changed. In this case, the elements of the covariance matrices were built on different correlations for each construct. The covariance matrix is a positively defined square matrix. Several tests with different correlations were developed until a model that meets the premises established in the initial model was found.

## 3. Results

Evaluation is performed based on the analysis of the initial proposals of the model. It is verified whether the results obtained through the relationship between latent variables support the conceptual proposal.

In terms of global adjustment, the Goodness-of-fit indexes of the initial models are not within the normally accepted boundaries. On the other hand, the calibrated model presents a suitable adjustment according to the thesis established by Anderson and Gerbing [47], Hair et al. [50], and Kline [44]. In this case, it can be confirmed that the calibrated model matches a representation, which is close to the population of interest. In terms of global adjustment, one can say that the Goodness-of-fit indexes are fit in the normally accepted limits. Table 2 shows the results of the confirmatory factor analysis for the models tested.

### 3.1. Cross Validation and Power Assessment

The elaboration of the cross-validation model, as the statistical control developed at this stage, is done in a way analogous to the previous model. In this sense, cross validation was formulated using the calibrated model. The results of the estimations regarding the significance level, the standardized solution and the values of *t* for each construct show significant results, confirming that the data used in the calibrated model corresponds properly to the initial models.

In order to contribute towards the validation of these results, a power assessment (potential for replication of study) was developed for the structure model, considering the prepositions established by Diamantopoulos [55]. The results of this analysis emphasized significant values for chi-square and degrees of freedom on the various testes conducted, offering strong reasons to believe that there are no serious discrepancies between the hypothetical model and the data. In other words, the tests conducted indicate that there are no serious discrepancies between the data obtained from the forms applied in the organizations accredited by the ONA accreditation methodology. Based on these results and the difference between the chi-square values for their respective degree of freedom (*x*^2^/df = 2.42), one can confirm that the data fit the model that evaluates the sustainability of accredited organizations.

### 3.2. Validation of the Sustainability Structural Model in Accredited Organizations

In this stage, some tests associated to the dimensionality, convergent validity, reliability, and discriminant validity were developed in order to validate the proposed model in this study. It can be observed from the statistical adjustments, Chi-square test (*X*^2^—Chi−square), the difference between chi-square and the degrees of freedom (*X*^2^/df), that the ratios GFI, RMR, SRMR, NFI, AGFI, PNFI, and RMSEA show results within the generally accepted limits and imply that the adjustments are acceptable. Evaluating the absolute values of standardized residuals, Table 3 shows that the results are not potential threats to the dimensionality of the model.

By taking the results of the extracted average variance, the factorial loads and the values of *t* > 2.58 obtained from the relation between the endogenous constructs (L) and the exogenous constructs (PM, C, QM, PO, S, and SU), one can verify that the model meets the established criteria for convergent validity [54]. The results shown in Table 4 demonstrate that the model fulfills the assumption of composite reliability considering the approach established by Hair et al. [50] and Marôco [43].

The results of the previous tests suggest support to discriminant validity. However, it can be verified, based on the analysis of the covariance matrix of the model, that most elements of the matrix have significant correlation. The tests performed in the Exploratory Factor Analysis (EFA) and Confirmatory Factor Analysis (CFA) were calculated based on the results of the covariance matrix, and these results are shown in Table 4.

**Figure 2:**
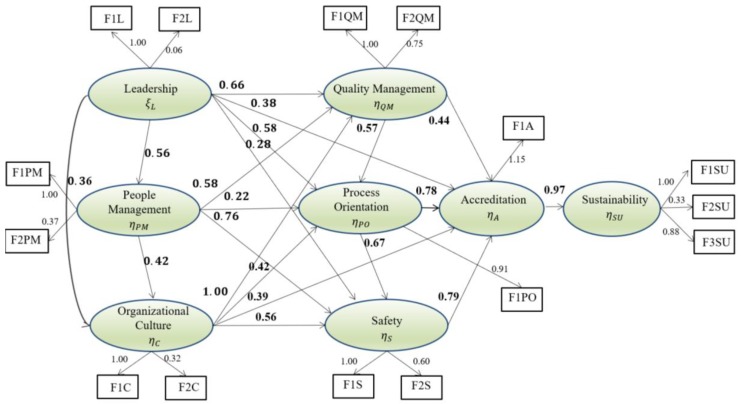
Standardized estimations of the calibrated model.

The models presented previously, initial and calibrated, were accepted after a series of tests performed to identify the model that fits the initial purpose. Figure 2 shows the standardized estimations of the calibrated model.

## 4. Discussion

Based on the results obtained for the three developed models, it can be concluded that the selected constructs can be used in the development of a structural model and to measure the ONA methodology.

In this process, the results show that leadership action is one of the most important factors in the process of health services accredited by ONA. Although, leadership, staff management, quality management, organizational culture, orientation for processes, and safety are strongly linked to the development of health organizations, and directly influence the accreditation process. These results harmonize with the studies developed by several authors such as Claver, Tarí, and Molina [13], El-Jardali et al. [30], Greenfield and Braithwaite [7], Scheirer [19], and Lee, Suh and Han [28].

The analysis of the following constructs, personnel management, organizational culture, quality management, orientation for processes, and safety and accreditation, were developed in a way analogous to the previous analysis. It can be stated that the personnel management construct has influenced in the hospital accreditation, in agreement with many thesis addressed in several countries, as Awuor and Kinuthia (10), Woo *et al.* (22), Li (24) and Fayer *et al.* (31).

By evaluating the influence of the organizational culture construct on the other constructs, it was possible to identify that the results of the initial model and calibrated model do not meet the expectations of the researches. On the other hand, it can be stated by analyzing the simulated model that organizational culture has a strong influence on the other constructs as reported by Awuor and Kinuthia [10], Boyer, Gardner, and Schweikhart [40], Faye et al. [31], and Woo et al. [22].

Regarding the quality management construct, only two relations were established for this study model, where what occurs in the initial model is not different from what was previously presented. On the other hand, the results of the three models show that quality management generally results in a significant influence on accreditation, a result consistent with several studies found in the literature as El-Jardali et al. [30] and Woo et al. [22].

It can also be stated that the orientation for process construct strongly influences accreditation, safety, and organizational sustainability—reinforcing what has been presented by Awuor and Kinuthia [10], Boyer, Gardner, and Schweikhart [40], Corrêa et al. [14], Kunkel, Rosenqvist, and Westerling [1], and Kunst and Lemmink [34]. The clinical organization accredited by the Brazilian methodology of hospital accreditation has been working on well-developed processes in all the tasks of the organization, especially in the areas of care.

The same occurs with the results obtained through CFA for the safety and accreditation constructs; the estimations obtained for the three proposals are strong and positive, especially in the simulated model. The results indicate that institutions accredited by the Brazilian accreditation methodology ONA have been working on issues related to safety, which have a relevant influence on the sustainability of accredited organizations.

### 4.1. Theoretical and Practical Implications

Based on the results obtained for the initial, calibrated and simulated models, as well as for the cross-validation model, it can be concluded that the selected constructs can measure the ONA methodology—which meets the objectives of this research.

The proposed model can be used by administrators in accredited organizations to evaluate the contribution factor of each construct within the context of accreditation and to focus efforts on factors that present low correlation and negatively interfere in the accreditation of the organization.

Contributions to health professionals are applicable to the evaluation of the personnel management construct, which can result in a major change in staff strategy, formally acknowledging its influence on service quality, task processes, organizational culture, accreditation itself and, consequently, on the sustainability of the organization. Contributions related to the Brazilian accreditation method go beyond the positive impact generated in accredited organizations. Based on the results of this research, it is possible to evaluate these constructs as well as their respective relations, proposing improvements in the handbook adopted by the methodology, including standards, procedures, methods, and techniques capable of enhancing the established relations. For accredited institutions, this research contributes to the evaluation process, since it is possible to better understand the influence of the relations proposed in this study. 

### 4.2. Limitations and Research Extensions

During the development of this research, it was possible to identify constraints that must be considered as opportunities for future studies. The difficulty to establish the existence of a strong correlation between the constructs of leadership and quality management (H1), leadership and orientation for processes (H3), personnel management and safety (H9), influence of organizational culture on quality management (H11), and organizational culture management and accreditation (H13), can be considered a constraint in this study. On the other hand, it was not possible to find a clear justification for the results obtained in the relationships established between organizational culture and accreditation (H13) and between quality management and accreditation (H15), which were refuted in the initial model. It can be concluded that the study could not effectively measure the quality management and organizational culture roles in the organizations accredited by the Brazilian accreditation methodology ONA, suggesting that these relations could be better investigated in future studies.

Further research may test different relationships between the factors selected in this study, involving hospitals with similar characteristics, such as their accreditation levels. In addition, future research could also verify the association between levels of accreditation and sustainability of organizations.

## 5. Conclusions

Some authors emphasize the importance of accreditation in health organizations, although few studies propose models that measure the accreditation methodology. By analyzing and validating the proposed measurement model, it can be verified that the selected factors satisfy the required criteria for the development of a structural model. This is confirmed by performing the confirmatory factorial analysis used to develop the model, where the relation between the previous variables were exhaustively investigated using the structural equation modeling technique, confirming the positive effects on accreditation.

Health organizations must incorporate the factors related to accreditation in their decisions, forcing themselves to rethink their priorities and plan more effectively. It should be not only a strategy of adopting habits, but also a compromise between all the members of different hierarchy levels in the organization. The study also concludes that leadership must work towards developing a quality program (quality management), which involves the creation of a motivated team (personnel management), improving processes (process orientation), and strengthening a safety program (safety), in order to develop an organizational culture focused on the best results regarding accreditation, and as a result, offering more sustainability to the organizations.

## Figures and Tables

**Table 1:** Confirmatory factor analysis (CFA) results.

Construct	Questions	Variables	Cronbach’s Alpha	Factors	Estimate (*γ*)	Standardized Solution (sd)	*t*-Value (*t*)	Communalities Sum of % Var
Leadership (L)	Q12	L1	0.8443	F1: Leadership performance	1.04	1.07	16.13	0.754
	Q13	L2	0.8455					
	Q14	L3	0.8394	F2: Leadership involvement	0.70	0.88	14.14	
	Q15	L4	0.8436					
	Q16	L5	0.8439					
	Q17	L6	0.8449					
Sustainability (SU)	Q18	SU1	0.8414	F1: Sustainable performance	1.00	0.91	1.00	0.785
	Q13	SU6	0.845					
	Q21	SU4	0.8482	F2: Commitment	0.82	0.92	8.58	
	Q22	SU5	0.8411					
	Q19	SU2	0.8428	F3: Achievement of goals	0.95	1.00	7.53	
	Q20	SU3	0.8398					
People Management (PM)	Q24	PM1	0.8393	F1: Information of professionals	1.00	0.83	9.83	0.770
	Q28	PM5	0.8427					
	Q25	PM2	0.8421	F2: Personal valuation	0.80	1.10	12.07	
	Q26	PM3	0.837					
	Q 27	PM4	0.8362					
Organizational Culture (C)	Q29	C1	0.8444	F1: Organizational Commitment	1.00	0.71	-	0.814
	Q31	C3	0.8389					
	Q30	C2	0.8402	F2: Performance	0.62	1.42	10.48	
	Q32	C4	0.8337					
	Q33	C5	0.8403					
Quality Management (QM)	Q34	QM1	0.8402	F1: Quality team involvement	1.00	0.71	-	0.769
	Q37	QM4	0.8384					
	Q38	QM5	0.8354					
	Q35	QM2	0.8472	F2: Quality Indicators	0.62	1.42	10.48	
	Q36	QM3	0.8407					
Process Orientation (PO)	Q39	PO1	0.8408	F1: Process orientation	1.00	0.82	-	0.820
	Q40	PO2	0.8441					
	Q41	PO3	0.8392					
	Q42	PO4	0.8407					
Safety (S)	Q43	S1	0.8404	F1: Safety culture	1.00	1.39	-	0.810
	Q44	S2	0.8396					
	Q45	S3	0.8367					
	Q46	S4	0.8383	F2: Risks	0.61	0.46	8.55	
	Q47	S5	0.8393					
Accreditation (A)	Q48	A1	0.8459	F1: Accreditation	0.88	0.60	5.63	0.865
	Q49	A2	0.8433					
	Q50	A3	0.8418					
	Q51	A4	0.8394					

**Table 2:** Adjustment measures for the model.

Index Type	Index Indicator	Initial Model Result	Calibrated Model Result	Simulated Model Result	Reference Value
Absolute Adjustment	X^2^—Chi-square	701.28	182.63	179.422	*p*-value > 0.05
	Degree of freedom (df)	75	77	74	Higher than one
	Normed Chi-square: X^2^/df	9.35	2.27	2.42	1 and 3: good Fit > 5 = bad
	Goodness-of-fit index (GFI)	0.777	1.212	0.968	≥0.90
	Root Mean Square Residual (RMR)	0.119	0.436	0.359	≤0.05
	Standardized Root Mean Residual (SRMR)	0.113	0.256	0.566	≥0.1
Incremental adjustment	Normed Fit Index (NFI)	0.749	0.939	0.925	≥0.9
	Comparative Fit Index (CFI)	0.767	0.967	0.927	≥0.9
Parsimonious adjustment	Adjusted Goodness of Fit Index (AGFI)	0.644	1.330	0.909	≥0.9
	Parsimony Normed Fit Index (PNFI)	0.535	0.835	0.875	Biggest value: best fit
	Parsimony Goodness-of-fit index (PGFI)	0.486	0.477	0.455	≤0.67 being 0.5 a good fit
Population adjustment	Root Mean square error of approximation (RMSEA)	0.170	0.412	0.494	0.03 to 0.08, being 0.05 a good fit

**Table 3:** Results obtained at the CFA of the structure model.

Structural Equations						
Relationships			Error Var	*R* ^2^	Composed Reliability	Extracted Variance
L	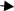	QM-A-PO-S-PM–C	0.90	0.66	0.90	0.66
PM	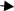	QM-PO–S-C	0.81	0.56	0.81	0.56
C	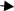	QM-PO-A-S	0.91	0.75	0.91	0.75
QM	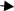	A–PO	3.15	0.54	3.15	0.54
PO	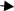	A–S	1.37	0.69	1.37	0.69
S	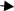	A	0.98	0.98	0.98	0.98
A	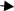	SU	1.07	1.07	1.07	1.07

**Table 4:** Covariance matrix for the latent variables.

Covariance Matrix for the Latent Variables						
	PM	C	QM	PO	S	A
PM	0.384					
C	0.396	0.777				
QM	1.119	0.906	5.935			
PO	0.583	0.477	2.068	1.000		
S	0.521	0.436	1.462	0.668	0.277	
A	0.537	0.435	2.407	0.927	0.656	1.000
SU	0.785	0.636	0.519	1.355	0.959	1.462
L	0.339	0.433	1.216	0.650	0.505	0.593

## Supplementary Materials

The following are available online at http://www.mdpi.com/1660-4601/15/11/2520/s1, S1: questionnaire.
