# Supplementary material for: Development of a System Measurement Model of the Brazilian Hospital Accreditation System
Source: Int J Environ Res Public Health. 2018 Nov 11;15(11):2520. doi: 10.3390/ijerph15112520 (PMC6266958; doi:10.3390/ijerph15112520)
Supplement: Supplementary file 1 [file ijerph-15-02520-s001.pdf]

Supplementary Materials - Questionnaire

Research on the Brazilian Accreditation Model

Part 1 - Identification

1. Name of the institution where you work:

2. What position do you hold in your organization?

3. How long have you been worked at the institution?
( ) 0 to 2 years ( ) 2 to 4 years ( ) Up to 4 years

4. In what state is the institution located?

5. What kind of service does your organization do:

( ) Ambulatory
( ) Hospital care
( ) Diagnostic Imaging, Radiotherapy and Nuclear Medicine
( ) Hemotherapy
( ) Hospital
( ) Laboratory
( ) Nephrology and Substitutive Renal Therapy
( ) Clothing Processing for Health Services
( ) Health Programs and Risk Prevention
( ) Ready care
( ) Diet Therapy Services
( ) Handling services

6. Size of organization - number of beds:
( ) Small: capacity less than 50 hospital beds;
( ) Medium: offers from 51 to 150 hospital beds;
( ) Large: offers from 151 up to 500 hospital beds;
( ) Special or extra cargo: it has more than 500 hospital beds;
( ) Other - no beds.

7. Type of organization:
( ) Public company
( ) Private company

8. Category:
( ) Nonprofit company
( ) Philanthropic company
( ) Nonprofit Benefit Company
( ) Beneficial For-Profit Company
( ) For-Profit Company

9. Number of employees in this organization:
( ) Up to 20
( ) 21 to 50
( ) 100 to 250
( ) 251 to 500
( ) Over 500.

10. Does the organization have teaching status?

( ) Yes
( ) No

11. Current Accreditation Level:
( ) Accredited
( ) Full accredited
( ) Accredited with excellence
( ) Certification seal
( ) Not accredited / In process of accreditation

Part 2 - Evaluating the company ...

12. The leadership team delegates activities to those who led and shares responsibilities.

I totally disagree	I strongly disagree	I disagree in parts	I do not agree or disagree	I agree in part	I strongly agree	I totally agree
(1)	(2)	(3)	(4)	(5)	(6)	(7)

13. The leadership team establishes the deadlines necessary to meet the quality goals for each employee.

I totally disagree	I strongly disagree	I disagree in parts	I do not agree or disagree	I agree in part	I strongly agree	I totally agree
(1)	(2)	(3)	(4)	(5)	(6)	(7)

14. The leadership team has the capacity to deal with mistakes and violations committed by employees, consolidating a justice culture.

I totally disagree	I strongly disagree	I disagree in parts	I do not agree or disagree	I agree in part	I strongly agree	I totally agree
(1)	(2)	(3)	(4)	(5)	(6)	(7)

15. The leadership team provides space for listening to divergent ideas and ideas that could make a difference in security processes.

I totally disagree	I strongly disagree	I disagree in parts	I do not agree or disagree	I agree in part	I strongly agree	I totally agree
(1)	(2)	(3)	(4)	(5)	(6)	(7)

16. The leadership team consistently uses the results of indicators in the decision-making process.

I totally disagree	I strongly disagree	I disagree in parts	I do not agree or disagree	I agree in part	I strongly agree	I totally agree
(1)	(2)	(3)	(4)	(5)	(6)	(7)

17. The leadership team evaluates the results of quality improvement actions incorporated in the organization with the participation of several authors.

I totally disagree	I strongly disagree	I disagree in parts	I do not agree or disagree	I agree in part	I strongly agree	I totally agree
(1)	(2)	(3)	(4)	(5)	(6)	(7)

18. The organization has a sustainable performance based on indicators.

I totally disagree	I strongly disagree	I disagree in parts	I do not agree or disagree	I agree in part	I strongly agree	I totally agree
(1)	(2)	(3)	(4)	(5)	(6)	(7)

19. The organization measures and assesses the risks associated with the activities performed.

I totally disagree	I strongly disagree	I disagree in parts	I do not agree or disagree	I agree in part	I strongly agree	I totally agree
(1)	(2)	(3)	(4)	(5)	(6)	(7)

20. The organization is committed to sustainability actions.

I totally disagree	I strongly disagree	I disagree in parts	I do not agree or disagree	I agree in part	I strongly agree	I totally agree
(1)	(2)	(3)	(4)	(5)	(6)	(7)

21. The organization periodically evaluates, and updates pre-established goals and objectives.

I totally disagree	I strongly disagree	I disagree in parts	I do not agree or disagree	I agree in part	I strongly agree	I totally agree
(1)	(2)	(3)	(4)	(5)	(6)	(7)

22. The organization establishes the management of clinical processes and these processes have a positive effect on financial results.

I totally disagree	I strongly disagree	I disagree in parts	I do not agree or disagree	I agree in part	I strongly agree	I totally agree
(1)	(2)	(3)	(4)	(5)	(6)	(7)

23. The organization develops its activities reducing the services and consumables' costs, as well as the preservation of their quality.

I totally disagree	I strongly disagree	I disagree in parts	I do not agree or disagree	I agree in part	I strongly agree	I totally agree
(1)	(2)	(3)	(4)	(5)	(6)	(7)

24. The organization develops and evaluates results of strategic people management indicators, proposing continuous improvement.

I totally disagree	I strongly disagree	I disagree in parts	I do not agree or disagree	I agree in part	I strongly agree	I totally agree
(1)	(2)	(3)	(4)	(5)	(6)	(7)

25. The organization has a leadership development and retention program.

I totally disagree	I strongly disagree	I disagree in parts	I do not agree or disagree	I agree in part	I strongly agree	I totally agree
(1)	(2)	(3)	(4)	(5)	(6)	(7)

26. The organization promotes professional development through a performance evaluation tool.

I totally disagree	I strongly disagree	I disagree in parts	I do not agree or disagree	I agree in part	I strongly agree	I totally agree
(1)	(2)	(3)	(4)	(5)	(6)	(7)

27. The organization uses information from professionals for decision-making and improvement.

I totally disagree	I strongly disagree	I disagree in parts	I do not agree or disagree	I agree in part	I strongly agree	I totally agree
(1)	(2)	(3)	(4)	(5)	(6)	(7)

28. The organization promotes actions for the identification and retention of knowledge.

I totally disagree	I strongly disagree	I disagree in parts	I do not agree or disagree	I agree in part	I strongly agree	I totally agree
(1)	(2)	(3)	(4)	(5)	(6)	(7)

29. The organization considers user satisfaction and this information has an impact on the performance of the organization.

I totally disagree	I strongly disagree	I disagree in parts	I do not agree or disagree	I agree in part	I strongly agree	I totally agree
(1)	(2)	(3)	(4)	(5)	(6)	(7)

30. The organization establishes a relationship between the organizational values and the strategic deployment of the organization.

I totally disagree	I strongly disagree	I disagree in parts	I do not agree or disagree	I agree in part	I strongly agree	I totally agree
(1)	(2)	(3)	(4)	(5)	(6)	(7)

31. The organization has the capacity to deal with errors and challenges.

I totally disagree	I strongly disagree	I disagree in parts	I do not agree or disagree	I agree in part	I strongly agree	I totally agree
(1)	(2)	(3)	(4)	(5)	(6)	(7)

32. The organization facilitates communication between the various levels of organization.

I totally disagree	I strongly disagree	I disagree in parts	I do not agree or disagree	I agree in part	I strongly agree	I totally agree
(1)	(2)	(3)	(4)	(5)	(6)	(7)

33. The organization implements patient-oriented actions and these actions are considered the essence of the business.

I totally disagree (1)	I strongly disagree (2)	I disagree in parts (3)	I do not agree or disagree (4)	I agree in part (5)	I strongly agree (6)	I totally agree (7)

34. The organization works with cycles of continuous improvement, monitoring of indicators and monitoring targets.

I totally disagree (1)	I strongly disagree (2)	I disagree in parts (3)	I do not agree or disagree (4)	I agree in part (5)	I strongly agree (6)	I totally agree (7)

35. The organization makes continuous use of quality indicators for quality measurement.

I totally disagree (1)	I strongly disagree (2)	I disagree in parts (3)	I do not agree or disagree (4)	I agree in part (5)	I strongly agree (6)	I totally agree (7)

36. The organization develops hospital performance indicators as a management tool.

I totally disagree (1)	I strongly disagree (2)	I disagree in parts (3)	I do not agree or disagree (4)	I agree in part (5)	I strongly agree (6)	I totally agree (7)

37. The organization has a strong role of committees and committees in monitoring the established goals and indicators.

I totally disagree (1)	I strongly disagree (2)	I disagree in parts (3)	I do not agree or disagree (4)	I agree in part (5)	I strongly agree (6)	I totally agree (7)

38. The organization identifies and evaluates the existence of non-conformities in the processes and procedures performed.

I totally disagree (1)	I strongly disagree (2)	I disagree in parts (3)	I do not agree or disagree (4)	I agree in part (5)	I strongly agree (6)	I totally agree (7)

39. The organization develops its care processes through a care line, reviewing the processes to correct its fragilities.

I totally disagree (1)	I strongly disagree (2)	I disagree in parts (3)	I do not agree or disagree (4)	I agree in part (5)	I strongly agree (6)	I totally agree (7)

40. The organization works with a critical analysis of process controls and results analysis.

I totally disagree (1)	I strongly disagree (2)	I disagree in parts (3)	I do not agree or disagree (4)	I agree in part (5)	I strongly agree (6)	I totally agree (7)

41. The organization establishes plans of action and improvements using processes and assistance protocols.

I totally disagree (1)	I strongly disagree (2)	I disagree in parts (3)	I do not agree or disagree (4)	I agree in part (5)	I strongly agree (6)	I totally agree (7)

42. The organization has work processes that act as barriers to the occurrence of adverse events.

I totally disagree (1)	I strongly disagree (2)	I disagree in parts (3)	I do not agree or disagree (4)	I agree in part (5)	I strongly agree (6)	I totally agree (7)

43. The organization has a committed patient safety core.

I totally disagree (1)	I strongly disagree (2)	I disagree in parts (3)	I do not agree or disagree (4)	I agree in part (5)	I strongly agree (6)	I totally agree (7)

44. The organization identifies and establishes actions to mitigate risks.

I totally disagree (1)	I strongly disagree (2)	I disagree in parts (3)	I do not agree or disagree (4)	I agree in part (5)	I strongly agree (6)	I totally agree (7)

45. The organization develops a plan of action for all incidents and adverse events that have been investigated.

I totally disagree	I strongly disagree	I disagree in parts	I do not agree or disagree	I agree in part	I strongly agree	I totally agree
(1)	(2)	(3)	(4)	(5)	(6)	(7)

46. In the organization, security is a set of tools to be implemented.

I totally disagree	I strongly disagree	I disagree in parts	I do not agree or disagree	I agree in part	I strongly agree	I totally agree
(1)	(2)	(3)	(4)	(5)	(6)	(7)

47. In the organization, security is solidly supported by a proactive culture.

I totally disagree	I strongly disagree	I disagree in parts	I do not agree or disagree	I agree in part	I strongly agree	I totally agree
(1)	(2)	(3)	(4)	(5)	(6)	(7)

48. The organization encourages waste reduction, process optimization, quality and technical assistance.

I totally disagree	I strongly disagree	I disagree in parts	I do not agree or disagree	I agree in part	I strongly agree	I totally agree
(1)	(2)	(3)	(4)	(5)	(6)	(7)

49. The organization promotes the reduction of problems caused by medical errors.

I totally disagree	I strongly disagree	I disagree in parts	I do not agree or disagree	I agree in part	I strongly agree	I totally agree
(1)	(2)	(3)	(4)	(5)	(6)	(7)

50. The organization establishes the evolution of the quality of the services provided after the accreditation process.

I totally disagree	I strongly disagree	I disagree in parts	I do not agree or disagree	I agree in part	I strongly agree	I totally agree
(1)	(2)	(3)	(4)	(5)	(6)	(7)

51. The organization has an environment where the employee feels more motivated by accreditation.

I totally disagree	I strongly disagree	I disagree in parts	I do not agree or disagree	I agree in part	I strongly agree	I totally agree
(1)	(2)	(3)	(4)	(5)	(6)	(7)
